# Impact of endometrioma management strategies on ovarian reserve over the follow-up period, a prospective longitudinal study

**DOI:** 10.3389/fendo.2025.1631108

**Published:** 2025-09-11

**Authors:** Kiper Aslan, Isil Kasapoglu, Bahadir Kosan, Tansu Bahar Gurbuz, Ludovico Muzii, Gurkan Uncu

**Affiliations:** ^1^ Bursa Uludag University School of Medicine, Department of Obstetrics and Gynecology, Bursa, Türkiye; ^2^ Department of Obstetrics and Gynecology, La Sapienza University, Rome, Italy

**Keywords:** endometrioma, anti-Mullerian Hormone, endometriosis, ovarian reserve, oral contraceptives (OCPs)

## Abstract

**Background:**

The effects of current treatment options for endometrioma on ovarian reserve remain controversial. Recent advancements in surgical techniques may challenge the previously established evidence regarding the detrimental effects of surgery on ovarian reserve. Additionally, whether medical suppression therapy provides a protective effect during this process remains an unanswered question. Furthermore, the impact on ovarian reserve in patients managed expectantly, without active intervention, is unclear.

**Objective:**

This study aims to evaluate the effects of endometrioma per se or its treatment modalities on ovarian reserve.

**Methods:**

In this prospective study, eighty women diagnosed with endometrioma via ultrasonography and twenty age-matched healthy women as controls were enrolled. The study group was divided into four subgroups, each consisting of twenty patients, based on the treatment modality received: expectant management, oral contraceptive pills (OCP), dienogest, and surgery. All participants underwent baseline ultrasonographic evaluations and blood sampling to determine serum anti-Müllerian hormone (AMH) levels at the time of enrollment. Follow-up assessments, including repeat ultrasonography and AMH measurements, were conducted six months after the initial evaluation.

**Results:**

The median six-month decline in serum AMH levels was 19% in the expectant management group, 26% in the OCP group, 21% in the dienogest group, 38% in the surgery group, and 8% in the healthy controls. Thus, statistically significant differences in AMH decline were observed between the OCP group and healthy controls (*p* = 0.034), and between the surgery group and healthy controls (*p* = 0.001).

**Conclusion:**

Despite advances in surgical techniques and precautions, surgical excision of endometriomas continues to pose a risk to ovarian reserve. Treatment with both dienogest and OCP is associated with a decrease in serum AMH levels, although the decline appears less significant with dienogest. Patients managed expectantly also showed a progressive decline in ovarian reserve compared to healthy controls.

**Trial Registration Number**

Clinical Trials, NCT03620838.

## Background

Endometriosis is a chronic gynecological disorder characterized by the presence of endometrial glandular and stromal tissue outside the uterine cavity (1). It is estimated to affect approximately 1.5% of women of reproductive age (2–4). The ovaries are the most commonly involved organs, and when endometriosis affects the ovaries, it typically presents as an ovarian endometrioma (OMA) (1). Studies suggest that approximately 40% of subfertile women with endometriosis are diagnosed with OMA (4–6).

Although a direct causal relationship between endometriosis and infertility has not been definitively established, infertility is a frequent complication among women with endometriosis, with the incidence of the disease ranging from 35% to 50% in the infertile population (1). Various stages of endometriosis can impair fertility by causing adhesions that distort pelvic anatomy, inflammation that alters endometrial receptivity, and inflammatory peritoneal fluid that disrupts sperm function (7–11). Additionally, ovarian reserve, which is a critical indicator of a woman's reproductive potential, may also be compromised.

OMA, the most common form of ovarian endometriosis, can be managed through medication, surgical intervention, or assisted reproductive technology (ART) in cases of infertility (12–14). For asymptomatic patients with favorable pelvic examination and ultrasonography, expectant management may be considered as a viable option (15, 16).

A key concern in the management of OMA is whether the disease itself or its treatment options—surgical or medical—has a more detrimental impact on ovarian reserve. Antral follicle count (AFC) and anti-Müllerian hormone (AMH) are widely used markers of ovarian reserve (17). However, to date, there is a limited body of research directly comparing the impact of conservative management versus surgical excision of OMA on ovarian reserve. Some studies have associated OMA itself with diminished reproductive potential, while others have shown similar findings for surgical excision of OMA (18). This makes it challenging to determine the optimal treatment approach for OMA, particularly in asymptomatic patients who may not wish to conceive immediately but are concerned about future fertility.

This longitudinal study aims to investigate the effects of OMA itself, managed expectantly, on ovarian reserve over time, and compare it with the effects of medical treatments such as oral contraceptive pills (OCPs) and dienogest, as well as surgical excision.

## Materials and methods

### Study protocol approval

The research ethics committee of Bursa Uludag University, a tertiary care university hospital, approved this prospective cohort study with the protocol number 2019-11/23. The study protocol was also registered at ClinicalTrials.gov (NCT NCT03620838).

### Patient enrollment

The study population was recruited from women of reproductive age who were not planning a pregnancy in the near future and had a serum AMH level >1.1 ng/mL. These women presented to our endometriosis clinic between March 2019 and January 2021. Eligible participants were diagnosed with endometrioma through ultrasonography, with cysts greater than 3cm in size, and a visual analogue scale (VAS) score >5 for associated pain. These patients were candidates for either medical or surgical treatment and were offered participation in the study.

The treatment groups consisted of:

- Expectant management (no intervention),
- Laparoscopic cystectomy, and
- Medical treatments, including oral contraceptive pills (OCP) or dienogest therapy.

The control group included healthy women of reproductive age with no ovarian or reproductive endocrine disorders. Pelvic ultrasonography was performed to rule out the presence of ovarian cysts in this group. To mitigate the possibility of undiagnosed superficial endometriosis, women with a history of primary dysmenorrhea, dyspareunia, or infertility were excluded from the control group.

Exclusion criteria for both the treatment and control groups included:

- Previous ovarian surgery,
- Irregular menstrual cycles,
- Reproductive endocrine disorders,
- Medical treatments (e.g., GnRH analogues) within the 6 months prior to recruitment that could potentially influence ovarian reserve markers,
- Any findings suggestive of malignancy.

### Treatment modalities

The choice of laparoscopic surgery was based on the presence of adenomyoma or deep infiltrating endometriosis (i.e., sacrouterine and vaginal nodule) and relatively high diameters of OMA. We performed the surgical technique as sharp dissection to identify the cleavage plane followed by blunt dissection and traction-countertraction for the stripping of the pseudocapsule. Then, we used laparoscopic scissors to break down the fibrotic adhesions between the pseudocapsule and the ovary. We avoided cauterization as far as possible and achieved hemostasis by suture where necessary. Any medical suppression approach was not used after surgical treatment.

Patients who refused or did not require surgery underwent medical treatment. The medication options were assigned depending on whether the patient demanded contraception. We prescribed OCP for these patients and oral dienogest 2mg/day for the rest.

The study's noninterference approach aimed to provide standardized and reliable data on endometriosis treatment options.

Patients with endometrioma >3cm in size not requiring medical or surgical treatment (i.e., no suspected ovarian cancer or organ involvement), minimal or no pain symptoms, and planned for occasional or intermittent nonsteroidal anti-inflammatory drug (NSAID) administration were offered to participate in the expectant management group.

### Ovarian reserve assessments

We measured serum AMH levels twice in all participants, once at recruitment and once at the 6-month follow-up visit, assessing the blood samples during the early follicular phase of the menstrual cycle by the "Beckman Coulter Access II" enzymatic-immunoassay. The detection limit of the test was ≤0.02 ng/mL. The rate of decline in serum AMH levels constituted the primary marker of ovarian reserve. Antral follicle count was not used to measure the ovarian reserve because of presence of OMA may obscure the exact AFC during ultrasonography.

### Statistical analyses

Data are presented as mean ± standard deviation (SD), median, and interquartile range (IQR), depending on the distribution characteristics. We used independent samples t-test or Mann-Whitney U test to compare continuous variables between the groups. In addition, we conducted subgroup analyses according to the laterality of endometriomas (i.e., unilateral or bilateral).

The rate of decline in serum AMH level for each patient was calculated by the formula (initial serum AMH level – serum AMH level at 6-month follow-up visit)/(initial serum AMH level) and expressed in percentage. We used correlation analysis to identify the factors associated with the rate of decline in serum AMH levels.

Given that the study includes only 20 participants per group, it may be underpowered to detect subtle inter-group differences or to perform robust multivariate modeling. To address this concern, a *post-hoc* power analysis was conducted to evaluate the statistical power of the study design. The power analysis was performed assuming a medium effect size and a two-tailed α of 0.05. The results of the power calculation indicated that the study design had 62.4% power to detect a significant difference in AMH decline between groups.

In addition, we performed a multivariate logistic regression analysis to isolate the independent effects of variables such as age, initial AMH level, cyst size, and laterality on the rate of AMH decline. A p-value < 0.05 was considered statistically significant for this analysis.

## Results

The study cohort consisted of 100 participants, including 20 women in each of the following groups: expectant management, OCP therapy, dienogest treatment, surgical intervention, and healthy controls. The mean (± SD) ages of the groups were 32.2 ± 6.9, 30.0 ± 6.3, 28.1 ± 4.4, 30.0 ± 6.2, and 32.1 ± 4.2 years, respectively. There was no statistically significant difference in age among the groups (*p* = 0.325).

The rate of unilateral endometriomas was 70% in the expectant management group, 63% in the OCP therapy group, 85% in the dienogest group, and 75% in the surgical intervention group, with no statistically significant difference between the groups (p = 0.56). The median cyst diameter and interquartile ranges (IQR) were as follows: 5 cm (IQR: 4–6) in the expectant management group, 5 cm (IQR: 4–7) in the OCP group, 4 cm (IQR: 3–5) in the dienogest group, and 10 cm (IQR: 8–12) in the surgery group. The difference in cyst size among the groups was statistically significant (*p* < 0.01) (**Table 1**).

**Table 1:** Demographic parameters and AMH results of the groups.

	Healthy	Expectant	Dienogest	OCP	Surgery	*p*
Age (S.D)	32.2 ± 4.8	32.2 ± 6.9	28.1 ± 4.4	30 ± 6.3	30 ± 6.2	0.33
Total Endometrioma Diameter* (cm)	N/A	5 (4-6)	4 (3-5)	5 (4-7)	10 (8-12)	<0.01^a^
Laterality Unilateral Bilateral	N/A	70% 30%	85% 15%	63% 37%	75% 25%	0.56
AMH (ng/mL) Initial*	4.7 (3.9-5.3)	3.2 (1.6-4.1)	3.4 (2.7-4.6)	3.0 (1.2-4.0)	2.2 (1.4-2.8)	<0.01^a^
AMH Six-month*	3.9 (3.0-5.6)	2.4 (1.3-3.5)	3.1 (1.3-4.6)	2.1 (0.3-3.2)	1.2 (0.8-2)	<0.01^b^
AMH Decline Rate*	-%8 (-25/15)	-19% (-38/-6)	-21% (-37/+7)	-26% (-66/-11)	-38% (-62/-32)	<0.01^c^

*Median values with 25–75 percentiles.

*Post-Hoc* analysis;

^a^Surgery group than the others p<0.01.

^b^Surgery, Expectant and OCP than the Healthy, respectively; p<0.001, p<0.02, p<0.023,

^c^Surgery and OCP than the Healthy, p<0.001, p<0.034.

At baseline, the median serum AMH levels (IQR) were as follows: Expectant management group: 3.2 ng/mL (IQR: 1.6–4.1), OCP therapy group: 3.0 ng/mL (IQR: 1.2–4.0), Dienogest group: 3.4 ng/mL (IQR: 2.7–4.6), Surgical intervention group: 2.2 ng/mL (IQR: 1.4–2.8), Healthy controls: 4.7 ng/mL (IQR: 3.9–5.3).

Among all groups, only the surgical group exhibited significantly lower baseline AMH levels compared to healthy controls (*p* = 0.001).

The median (IQR) serum AMH levels at the 6-month follow-up visit were 2.4 (1.3-3.5) ng/mL, 2.1 (0.3-3.2) ng/mL, 3.1 (1.3-4.6) ng/mL, 1.2 (0.8-2) ng/mL, and 3.9 (3-5.6) ng/mL in the groups, respectively.

At the 6-month follow-up, a significant decline in AMH levels was observed in the expectant management, OCP, and surgical groups compared to the healthy control group. In contrast, no statistically significant reduction in AMH levels was detected in the dienogest group. (*p* = 0.023, *p* = 0.02, and *p*<0.001, respectively).

**Figure 1:**
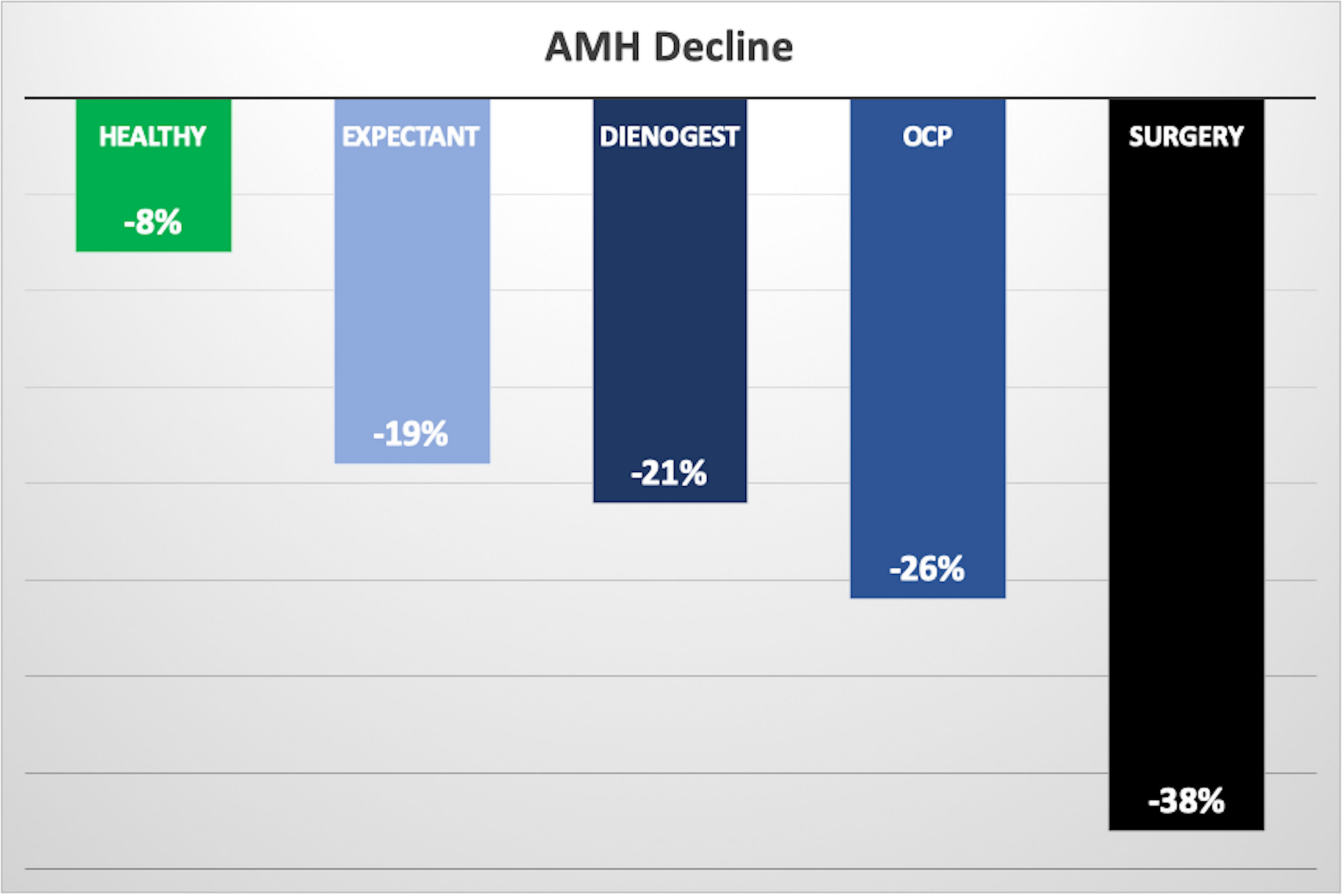
Six Months AMH decline of the endometrioma treatment groups vs. healthy controls, with median percentages.

The percentage reductions in AMH levels over six months for each group were as follows: 19% (from 3.8 to 3.1 ng/mL) in the expectant management group, 26% (from 6.6 to 4.9 ng/mL) in the OCP therapy group, 21% (from 3.7 to 2.9 ng/mL) in the dienogest group, 38% (from 6.2 to 3.2 ng/mL) in the surgical group, and 8% (from 2.5 to 2.3 ng/mL) in the healthy controls. The most pronounced decline was observed in the surgical group. The reduction noted in the healthy control group is considered part of the expected physiological decline. When compared with the healthy controls, the AMH reductions in both the OCP (*p* = 0.034) and surgical (*p* = 0.001) groups were statistically significant (**Table 1**, **Figure 1**).

A correlation analysis was conducted to determine whether any parameters were associated with the rate of AMH decline. The results indicated that both patient age and initial AMH levels were significantly correlated with the decline in AMH (**Table 2**). Moreover, multivariate logistic regression analysis identified age as the only significant predictor of AMH decline when compared with cyst laterality, baseline AMH levels, and cyst diameter (**Table 3**).

**Table 2:** Correlation analysis of the variables.

Spearman correlation	Correlation coefficient	*p*
	AMH decline rate	
Age	-0.226	0.03
Laterality	-0.136	0.29
Cyst Diameter	0.258	0.08
AMH - Initial	-0.221	<0.01

**Table 3:** Multivariate logistic regression analysis.

	St. Beta	St. Error	95 CI
Age	-0.378	0.04	-0.057 – 0.0
Laterality	-0.032	0.8	-0.47 – 0.41
AMH Initial	-0.278	0.17	-0.17- 0.03
Cyst Diameter	0.136	0.9	-0.09 – 0.99

## Discussion

Ultrasonography currently serves as the primary diagnostic modality in the presence of endometrioma. Among endometriosis subtypes, endometrioma is more readily identifiable due to its characteristic ultrasonographic features, thereby facilitating the diagnostic process. However, the approach to managing an endometrioma after diagnosis varies significantly, influenced by several clinical factors including the patient's symptoms, age, desire for fertility, cyst size, and the presence of comorbidities such as pelvic pain or other forms of endometriosis. For patients who do not require immediate infertility treatment, management options typically fall into three main categories: expectant management, surgical intervention, and medical therapy (19). Among medical therapies, the two principal options are the use of OCP or progestins (20).

The course of serum AMH levels—one of the key indicators of ovarian reserve—during the management of endometrioma remains a subject of ongoing debate.

This study was designed to evaluate the course of serum AMH levels during the treatment process by comparing four different management strategies applied in the presence of endometrioma, including a comparison with a healthy control group.

Four major conclusions were drawn from this comparative evaluation.

The first key finding demonstrated a reduction in ovarian reserve among patients with endometrioma compared to healthy controls. Although not statistically significant, our results indicated a numerically greater but not statistically meaningful decline in serum anti-Müllerian hormone (AMH) levels in the endometrioma group (19%) relative to the control group (8%) (*p* = 0.65).

Ovarian reserve is a critical component of female reproductive potential and any intervention planned after the ultrasonographic identification of endometrioma must be assessed in terms of its potential impact on ovarian reserve, and patients should be adequately informed.

Previous meta-analyses have shown that untreated endometriomas are significantly associated with lower serum AMH levels (21).

Similarly, our earlier study on endometrioma-related ovarian reserve reduction (ERROR study) suggested that women with endometrioma exhibit a more rapid and progressive decline in AMH levels compared to healthy individuals (22).

The impact of endometrioma on ovarian reserve is hypothesized to occur through two main mechanisms: First mechanical compression of the ovarian cortex, leading to impaired blood flow, and second, inflammatory responses within endometriotic lesions, resulting in follicular damage. While the difference in the rate of AMH decline between the endometrioma and control groups did not reach statistical significance, a progressive reduction was consistently observed in the endometrioma cohort.

To minimize potential bias due to low baseline AMH levels and to avoid the "burn-out" effect resulting from a depleted primordial follicle pool, the present study included only women with normal initial AMH levels (>1.1 ng/mL) (23). This methodological choice allowed for a more accurate assessment of the rate of decline in ovarian reserve.

Given these findings, patients with endometrioma managed expectantly should be closely monitored. Even in cases where baseline ovarian reserve appears within normal limits, patients should be counseled regarding the potential for future decline and undergo regular follow-up assessments to evaluate ovarian function.

The second major finding of this study pertains to the impact of surgery for endometriomas on ovarian reserve.

Although numerous earlier studies have documented the detrimental effects of endometrioma surgery on ovarian reserve, much of this evidence is based on older data.

Advances in surgical techniques, the use of equipment that minimizes thermal damage, adherence to principles such as avoiding excessive coagulation, and implementation of ovary-sparing strategies may have altered these outcomes in recent years.

Two systematic reviews have reported a significant decline in serum AMH levels following surgical excision of endometriomas (24, 25). More recent studies have also confirmed reductions in ovarian reserve after surgery, with approximately a 30% decrease in AMH levels following unilateral cystectomy and up to 44% after bilateral procedures. This reduction may occur at various stages of the surgical process, including the removal of healthy ovarian cortex containing primordial follicles and the use of thermal coagulation for hemostasis. Recent systematic reviews have emphasized that the choice of hemostatic technique significantly influences the extent of AMH decline. Energy-based modalities (such as cauterization and laser techniques) tend to result in the most considerable damage to ovarian tissue and a pronounced decrease in AMH levels. In contrast, suturing the ovary to achieve hemostasis has been associated with less harm to ovarian reserve (26, 27). Collectively, the available evidence suggests that surgical excision of endometriomas can result in a permanent reduction in ovarian reserve.

Several studies have noted that endometriomas larger than 5cm in diameter are associated with a more significant decline in AMH levels (28). However, other studies have found no relationship between cyst size and AMH decline, although many of these studies included relatively small sample sizes (29, 30). A recent meta-analysis concluded that the excision of bilateral endometriomas and cysts greater than 7cm in diameter is associated with a more pronounced decline in AMH levels. The mechanical removal of healthy ovarian tissue and inflammatory damage to the cortex may account for these findings (31).

In our study population, the greatest decline in AMH levels (38%) was observed in the surgical treatment group, consistent with findings in the current literature. It should be noted, however, that patients in the surgical group had significantly larger median cyst diameters compared to those in the other groups. Therefore, the observed reduction in AMH may be partially attributed to larger OMA sizes and already low baseline AMH levels among patients requiring surgery. On the other hand, our correlation and logistic regression analyses showed that cyst diameter had no significant impact on AMH decline, suggesting that factors other than cyst size may have contributed more significantly to the observed changes in ovarian reserve.

At present, it appears unlikely that endometrioma surgery can be performed without causing some degree of damage to ovarian reserve. However, this damage may be minimized depending on the surgeon's level of experience (32).

The third major finding of the study is that serum AMH levels remained similar in patients treated with dienogest compared to those who were managed with expectant follow-up without any medical intervention.

Although the role of medical treatment in the management of endometriosis-associated infertility has long been regarded as limited, hormonal suppression remains the first-line treatment option for addressing pain related to endometriosis (33, 34).

Traditionally, OCP have been recommended as the first-line agents for medical suppression; however, in recent years, progestins—particularly dienogest—have largely replaced OCP in this role. Although no significant differences in efficacy have been observed when compared with other progestins (35), dienogest remains the most commonly used progestin for the medical suppression of endometriosis today.

Although dienogest has been shown to be effective in numerous clinical studies, data on its impact on ovarian reserve remain limited.

The only study that specifically evaluated the effect of dienogest on ovarian reserve is that by Takenaka et al. (36), which included 30 patients who were administered either dienogest or leuprolide for 12 weeks prior to surgery. While the authors did not present detailed comparative data for each group, they reported no significant change in serum AMH levels across the entire cohort. Similarly, the Visanne Post-approval Observational Study (VIPOS)—the largest study conducted to date on the safety of dienogest and other hormonal therapies—did not evaluate ovarian reserve (37). Thus, current evidence on serial changes in ovarian reserve markers during dienogest therapy for endometrioma remains insufficient.

A more recent prospective study by Muzii et al. (20) investigated the impact of a 6-month course of dienogest on ovarian reserve and found no significant change in AMH levels (a decrease of 18%) among 32 patients with endometrioma, which is consistent with our findings.

In our study, no significant difference in the 6-month decline in AMH levels was observed between patients managed expectantly and those receiving dienogest. However, in a comparable group of patients treated with combined oral contraceptive pills, a 26% reduction in AMH levels was observed. The observed difference between dienogest and OCP is not strong enough to support a definitive conclusion that dienogest is superior to OCP in preserving ovarian reserve. Dienogest, a progestin with anti-inflammatory properties, works by inhibiting the growth of endometriotic lesions and suppressing ovarian activity, yet its mechanism of action appears to have a less detrimental impact on ovarian reserve than OCPs. OCPs suppress gonadotropin release and, consequently, reduce follicle-stimulating hormone (FSH) levels, which can lead to a more significant suppression of folliculogenesis and a reduction in ovarian reserve markers like AMH. On the other hand, dienogest, while also suppressing ovarian function, may exert a more localized effect on the endometriotic lesions rather than a systemic suppression of ovarian activity, potentially explaining its less pronounced effect on AMH levels. A more accurate answer to this question will require the evaluation of AMH levels following the discontinuation of treatment. Nevertheless, the continuous use of dienogest, which may lead to more sustained suppression, as well as its anti-inflammatory properties demonstrated in the literature, may represent theoretical explanations for this difference (38–40).

The final key finding of this study pertains to the impact of OCP use on ovarian reserve. OCP remain one of the most widely used medical treatments for endometriosis. Importantly, the choice of OCP in endometriosis management is not necessarily tailored to the disease itself, but rather, any formulation containing progestins is generally considered sufficient. This preference stems not from the intrinsic effects of progestins, but rather from the suppressive impact of OCPs on gonadotropin release, which can influence ovarian activity.

In our study, a 26% reduction in serum AMH levels was observed after 6 months of OCP use. This decline is likely transient rather than permanent. OCP suppress the secretion of follicle-stimulating hormone (FSH), thereby inhibiting FSH-dependent folliculogenesis. Since FSH is essential for the development of preantral and early antral follicles that produce AMH, this suppression may account for the observed decline (41, 42). Several studies suggest that the suppressive effect of hormonal contraception on AMH levels is reversible within 3 to 6 months following discontinuation (43, 44). The reduction in the total number of antral follicles, the shift toward smaller follicle size, and gonadotropin suppression likely contribute to this phenomenon.

One of the most important questions emerging from this study is how the decline in serum AMH levels observed during progestin and OCP treatment behaves following the discontinuation of therapy. Specifically, it remains unclear whether these changes persist, reverse, or normalize when compared to both untreated patients with endometrioma and healthy individuals without the disease. Addressing this question is critical for understanding the long-term implications of medical suppression on ovarian reserve and for determining whether the observed decline is a reversible pharmacologic effect or a sustained biological alteration. Future longitudinal studies with post-treatment follow-up are warranted to clarify the trajectory of AMH levels across different management strategies.

An important point to highlight is that patient age emerged as the strongest predictor of AMH decline in the regression analysis, surpassing other factors such as initial AMH levels, cyst laterality, and cyst size. Therefore, age should be carefully considered when planning and guiding treatment strategies for endometrioma. And also, the clinical relevance of the differential AMH declines observed in this study (e.g., 19% vs. 38%) warrants further discussion, especially in terms of its potential implications for fertility outcomes. A more significant decline in AMH levels, as seen in the surgery group (38%), could suggest a higher risk of reduced ovarian reserve and diminished fertility potential, whereas a smaller decline (e.g., 19% in the expectant management group) may indicate a relatively less detrimental effect on ovarian reserve. These findings highlight the importance of considering both treatment modalities and the degree of AMH decline when counseling patients about their fertility prospects.

## Strengths and limitations

Strengths of our study include its prospective cohort design, the use of a healthy control group, and the inclusion of multiple treatment modalities for endometrioma. This study has certain limitations. The most notable is the relatively small sample size and the non-randomized design, which may restrict the generalizability of the results. Randomization in studies of this nature is challenging due to the involvement of multiple groups and the difficulty in recruiting patients with OMA, who are often reluctant to participate in studies that could potentially affect their future fertility. The non-randomized design led to differences in initial AMH levels between groups; however, since the primary focus was on the rate of AMH decline, these baseline differences are unlikely to have introduced bias, as regression analysis demonstrated that initial AMH levels had no effect on the rate of decline.

Given the monocentric nature of our cohort and the exclusion of ART-seeking women, the generalizability of our results may be limited. Future multicentric studies with larger cohorts are necessary to validate these results. Furthermore, the absence of long-term postoperative follow-up limits our ability to determine whether the observed declines in AMH levels are permanent or reversible, and whether medical treatment can support fertility preservation in the long term.

Ovarian reserve in this study was assessed solely based on serum AMH levels, without the inclusion of other markers such as FSH or antral follicle count (AFC). Nonetheless, given that endometriomas and other cysts can obscure sonographic visualization of antral follicles, AMH remains the most reliable marker in these cases. Although AMH is a systemic and non-lateralized marker, it has been shown to provide a consistent and objective measure of ovarian reserve, particularly useful in evaluating the impact of surgical interventions (18).

## Conclusion

In conclusion, despite advances in surgical techniques and precautions, surgical excision of endometriomas continues to pose a risk to ovarian reserve. Treatment with both dienogest and OCP is accompanied by a decrease in serum AMH levels, although the decline appears to be less significant with dienogest. Patients managed expectantly also demonstrated a progressive decline in ovarian reserve compared to healthy controls. Therefore, even in women with initially normal ovarian reserve, close monitoring is recommended in the presence of endometriosis, particularly in those of advanced reproductive age.

## Data Availability

The raw data supporting the conclusions of this article will be made available by the authors, without undue reservation.
